# Supplementary material for: Timing of Transfusion, not Hemoglobin Variability, Is Associated with 3-Month Outcomes in Acute Ischemic Stroke
Source: J Clin Med. 2020 May 21;9(5):1566. doi: 10.3390/jcm9051566 (PMC7290978; doi:10.3390/jcm9051566)
Supplement: Supplementary file 1 [file jcm-09-01566-s001.pdf]

Table S1. The comparison between patients with and without red blood cell transfusion during the admission.

	**Transfusion (*n* = 132)**	**Non-transfusion (*n* = 2566)**	**Total (*n* = 2698)**	***p***
Age, year	71.6±13.5	68.0±13.1	68.2±13.1	<0.001
Male	61(46.2%)	1,521(59.3%)	1,582 (58.6)	0.004
Past medical history				
Stroke	45 (34.1%)	565 (22.0%)	610 (22.6%)	0.002
Hypertension	93 (70.5%)	1,707 (66.5%)	1,800 (66.7)	0.401
Diabetes	40 (30.3%)	888 (34.6%)	928 (34.4)	0.357
Hyperlipidemia	38 (28.8%)	838 (32.7%)	999 (32.5)	0.406
Current smoking	31 (23.5)	861 (33.6)	892 (33.1)	0.021
previous mRS, score	0 (0-2)	0 (0-0)	0 (0-0)	<0.001
Stroke subtype				<0.001
LAA	41 (31.1%)	886 (34.5%)	927 (34.4%)	
SVO	6 (4.5%)	787 (30.7%)	793 (29.4%)	
CE	39 (29.6%)	514 (20.0%)	553 (20.5%)	
SOE	9 (6.8%)	59 (2.3%)	68 (2.5%)	
SUE	37 (28.0%)	320 (12.5%)	357 (13.2%)	
NIHSS, score	10 (3-17)	3 (1-7)	3 (1-8)	<0.001
Thrombolysis	16 (12.1%)	264 (10.3%)	280(10.4%)	0.598
onset to visit time, hour (IQR)	3.7 (1.1-29.7)	8.0 (1.6-34.6)	7.8 (1.6-34.5)	0.057
Laboratory parameter				
WBC, $10^3/\mu L$	9.3±4.3	8±2.8	8.1±2.9	<0.001
Platelet, $10^3/\mu L$	255±134	234±77	236±81.1	0.005
BUN, mg/dL	22.3±16.2	16.9±7.8	17.2±8.5	<0.001
Creatinine, mg/dL	1.2±1.2	1±0.9	1±0.9	0.004
Total cholesterol, mg/dL	153.7±49.3	176.1±42.9	175±43.5	<0.001
TG, mg/dL	104.5±55.7	126.5±81.5	125.4±80.5	0.003
HDL, mg/dL	43.7±12.5	46.7±12.7	16.6±12.7	0.009
LDL, mg/dL	91.5±41.8	109.3±36.2	108.5±36.7	<0.001
FBS, mg/dL	136.1±56.5	124.6±52.4	125.1±52.7	0.014
INR	1.2±0.8	1.0±0.3	1.1±0.3	<0.001
Systolic BP, mmHg	140.6±26.6	144.4±25.4	144.2±25.5	0.1
Diastolic BP, mmHg	80.5±15.8	83.6±14.5	83.5±14.6	0.016
History of antiplatelet agent usage	45 (34.1%)	788 (32.9%)	833 (30.9%)	0.469
History of anticoagulation usage	13 (9.8%)	125 (4.8%)	138 (5.1%)	0.02

History of antithrombotics usage	56 (42.4%)	883 (34.4%)	939 (34.8)	0.073

Categorical variables are represented by the number (frequency percent), and continuous variable are represented by the mean (± standard deviation) or median (interquartile range) as appropriate. mRS, modified Rankin Scale; NIHSS, National Institute of Health Stroke Scale; WBC, white blood cell, BUN, blood urea nitrogen; TG, triglycerides; HDL, high-density lipoprotein, LDL, low-density lipoprotein; FBS, fasting blood sugar; INR, international normalized ratio; BP, blood pressure.

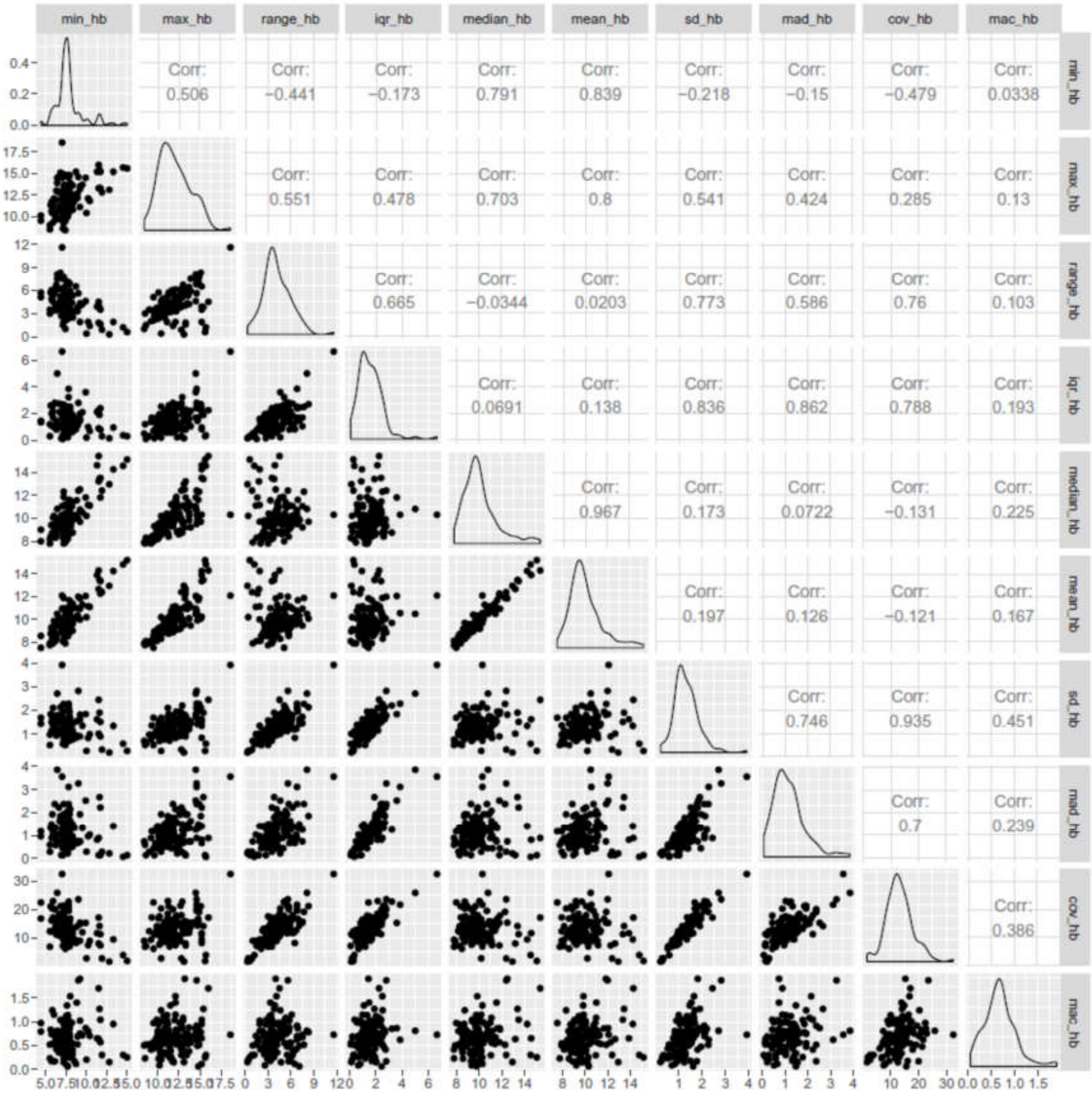

Figure S1. The correlation between hemoglobin variability parameters. iqr: interquartile range; sd: standard deviation; mad: median absolute deviation; cov: coefficient of variance, mac: mean absolute change.
